# Retained transorbital foreign body with intracranial extension after pipe bomb explosion

**DOI:** 10.4103/2152-7806.74241

**Published:** 2010-12-25

**Authors:** Ekkehard M. Kasper, Markus M. Luedi, Pascal O. Zinn, Peter A.D. Rubin, Clark Chen

**Affiliations:** Department of Neurosurgery, Beth Israel Deaconess Medical Center, Harvard Medical School, Boston, MA, USA; Department of Ophthalmic Plastic and Orbital Surgery, Brookline, MA; University of Tennessee, USA

**Keywords:** Brain injury, explosion, intracranial foreign body, pipe bomb, transorbital

## Abstract

**Background:**

Penetrating brain injuries caused by explosions are survived in extremely rare cases only. However, potential casualties of such cases may be encountered by regular physicians even outside a war zone, e.g., due to an assault or terror blast. There is very limited literature to this end; therefore, we report the successful neurosurgical management of a penetrating head injury due to a pipe bomb explosion.

**Case Description:**

A 19-year-old man was brought to the ER with a swollen, bleeding right orbit, and a severely injured left hand after having sustained an unwitnessed explosion from a self-made pipe bomb. He presented with a GCS (Glasgow Coma Scale) of 15 at time of admission, work-up revealed an intracranial retained metal fragment measuring 5 × 1 × 0.2 cm lodged retro-orbitally and in the skull base. The patient underwent emergent right temporal craniotomy and temporal lobectomy and simultaneous right enucleation before the petrous bone and sphenoid wing lodged metal fragment was successfully removed.

**Conclusion:**

This case underscores the importance of having a high suspicion for the presence of an intracranial injury and a retained foreign body in the setting of a penetrating head injury. Aggressive and timely workup as well as expeditious surgical management are crucial in these settings and can generate exceptionally good outcomes despite a major trauma.

## INTRODUCTION

Currently, in the United States and Europe with a yearly average of Incidence of 250/100 000 cases, head trauma accounts for the majority of trauma deaths and most penetrating brain injuries result from gun shots, either self-inflicted or related to armed conflicts and warfare.[15]

Explosions are a leading cause of traumatic brain injury among active duty military personnel in war zones. Veterans’ advocates believe that 10%–20% of Iraq veterans have some level of traumatic brain injury (TBI). Civilian gunshot wounds were shown to have a fatality rate of over 90%–95%. Beyond this, only an extremely small fraction of penetrating brain injuries due to explosions is survived.[2,7]

The ability of an object to penetrate the bone and to enter the intracranial cavities is determined by a number of variables: energy, features of the object (tip shape, velocity), and angle of approach. After the initial impact, which causes separation and cavitation, all damage in the target tissue is caused by the radially redirected kinetic energy of the expanding tissue itself.[6] Sir Victor Horsley, one of the earliest pioneers in neurosurgery, showed as early as 1915 that a traumatic cavity reaches 10–40 times the size of the offending projectile.[5] Low energy penetrating injuries are a rare entity and patients with severe high energy head injuries and an abnormal head CT and/or a compound or skull base fracture have a tenfold higher mortality than trauma patients without such injuries.[10] The majority of patients suffering from penetrating civilian gunshot wounds and admitted with GCS <9 will not survive.[9] An 8% rate of infection or abscess formation after penetrating cranio-cerebral gunshots does not significantly influence the outcome in a retrospective review from 1996 to 2003.[9] Studies of cranio-cerebral gunshot victims demonstrate a mortality twice as high as in patients after road accidents and threefold higher than other penetrating head traumas.[1,11]

Here we present the management and recovery from a penetrating brain injury with a retained intracranial foreign metal object in a young man mishandling a self-made pipe bomb.

## CASE PRESENTATION

### History and Examination

**Figure 1:**
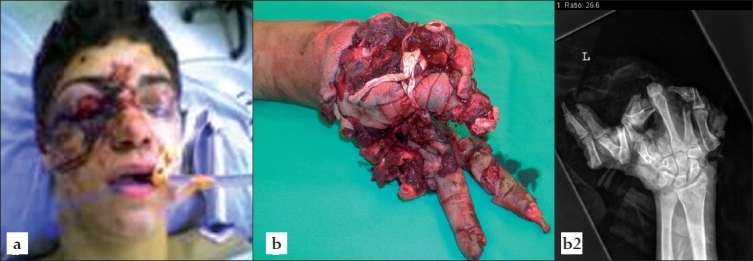
Preoperative films showing the external appearance of a 19-year-old man admitted for an unwitnessed explosion: Note the rather minimal facial laceration (a) but significantly severed left hand (b).

A 19-year-old man without significant past medical history was brought to the Emergency Department having been found unconscious at home, after an explosion of unclear origin was noticed in the basement of the family home. Upon admission, the patient was fully conscious and stated, that he had been handling a self-made pipe bomb that exploded unexpectedly, severely injuring his left hand and “somehow hitting his right side of the face.” At the scene, he complained about a headache and the inability to see with his right eye, which was blood crusted. The patient also reported that he could not feel his left hand, which was severely injured. On presentation to the ER (trauma level 1 institution), he was stable and conversant with a GCS of 15 with minimal residual bleeding from his face and a profusely bruised and swollen right orbit [Figure 1a]. His left hand was partially amputated [Figure 1b]. The patient subsequently underwent primary and secondary surveys according to ATLS protocol and was sent for imaging studies. An ophthalmology consult was obtained in the ER, which revealed gross destruction of the right eye with globe rupture and no residual vision. His contralateral left globe was fully intact the only neurological abnormality was progressive drowsiness, which led to expeditious imaging work-up.

### Neuroimaging Findings

**Figure 2:**
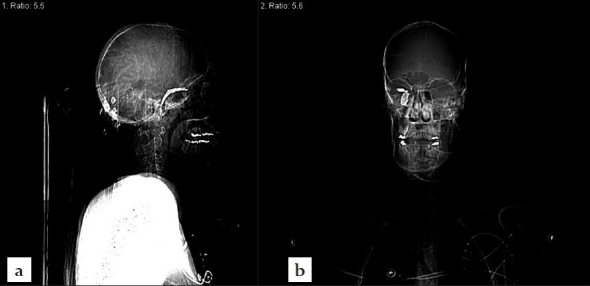
CT scout images demonstrating an intracranially retained foreign body. (a) Lateral view. (b) A/P view.

**Figure 3:**
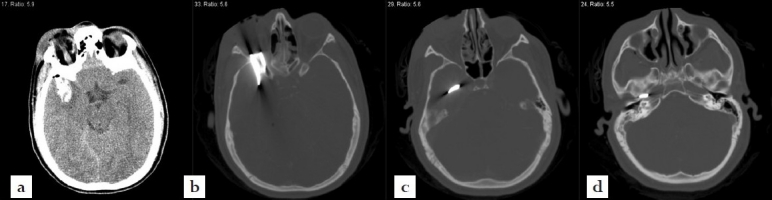
Axial noncontrast CT scans of the head (a) soft tissue window and (bd) bone window) demonstrating the retained metal fragment measuring ca. 5 cm × 1.5 cm × 0.2 cm.

**Figure 4:**
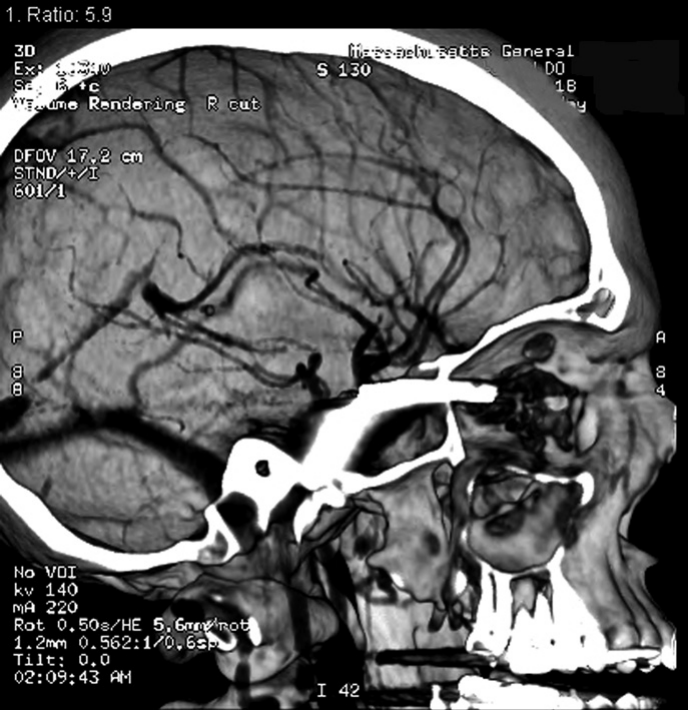
Sagittal contrast enhanced CTA scans demonstrating the retained metal fragment measuring as it is situated from the retro-orbital wall and curves through the temporal lobe into the skull base.

Initially a noncontrast CT scan of the head was performed emergently and revealed an intracranial irregularly shaped metal fragment measuring approximately 5 × 1.5 × 0.2 cm as seen on the tomogram scout films [Figure 2]. The object was lodged in the back of the right orbit, reached through the superior orbital fissure into the middle cranial fossa and transversed the right temporal lobe. It was comparable to a near complete blow-out fracture of the right orbit with adjacent ethmoid- and sphenoid-fractures before the fragment penetrated posteriorly into the floor of the middle cranial fossa formed by the petrous portion of the right temporal bone and skull base [Figure 3]. For further analysis, a CT angiography was performed revealing the tip of the metal fragment had penetrated the petrous bone deeply reaching the right internal carotid canal [Figure 4].

### Treatment and Outcome

While examinations were ongoing, the patient became progressively restless and drowsy. He was emergently intubated in the ER and taken to the OR for neurosurgical exploration in conjunction with an orthopedic trauma team who simultaneously took care of the patient’s left hand. The patient received intravenous antibiotics (vancomycin 1 g, flagyl 500 mg, gentamycin 80 mg) and a tetanus vaccination. For brain relaxation 100 g I/V Mannitol and 20 mg I/V lasix with mild hyperventilation (*p*CO_2_ = 30) was administered along with 10 mg of I/V Decadron for a possible temporal lobectomy. Two units of fresh frozen plasma were infused to prevent DIC and other blood products were readily available.

**Figure 5:**
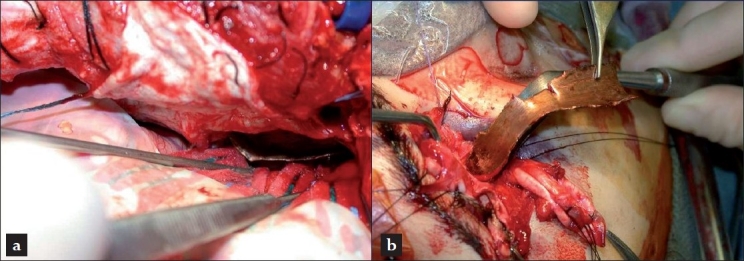
Intraoperative situs (a) intracranial view from above into the middle cranial fossa with cottonoids retracting the resection margin of the temporal lobe and (b) view onto the right supraorbital ridge and orbit during evacuation of the foreign body. (a) Intracranial view. (b) View of the orbit after enucleation during removal of the metal.

Surgical exposure was accomplished via a right fronto-temporal craniotomy and simultaneous right neck exposure of the right common carotid artery, to allow for proximal control in case of significant intracranial hemorrhage. A right temporal lobectomy was carefully performed around the retained foreign body and the skull base was explored. The copper pipe fragment [Figure 5a] which was firmly lodged into the petrous bone and sphenoid wing was exposed. The part of the metal fragment in the petrous bone was safely loosened from the intracranial side. An occuloplastics consult was performed intraoperatively. As the eye was severely and irrepairably injured, a right-sided enucleation was performed. This procedure also facilitated further transorbital exploration and allowed to mobilize the metal fragment which was lodged in the sphenoid. After drilling down parts of the orbital apex and sphenoid wing around the metal fragment, which reached the floor of the middle cranial fossa, it was possible to mobilize and retrieve the entire foreign body in one single piece via the transorbital route[3,4] [Figure 5b]. Simultaneous packing of the petrous portion of the temporal bone was performed. As a precaution, we had exposed and slinged the ICA at the level of the CCA bifurcation to allow for possible ICA occlusion in the neck, should the packing be unsuccessful. During careful exploration, no laceration of the intracanalicular ICA was evident and the skull base defect was closed. The floor of the middle cranial fossa and the retro-orbital wall was closed with a titanium mesh (Synthes) and an onlay duroplasty allograft (Duragen; Integra). Due to the severity of the injury, we felt safer to leave the patient with a decompressive hemicraniectomy.

**Figure 6:**
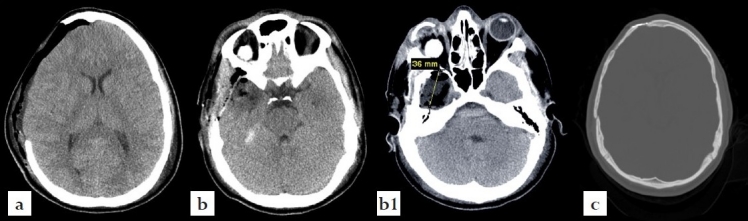
Postoperative CT scans of the brain (a) post-hemicraniectomy, (b) temporal lobectomy, and (c) after second stage autologous cranioplasty.

**Figure 7:**
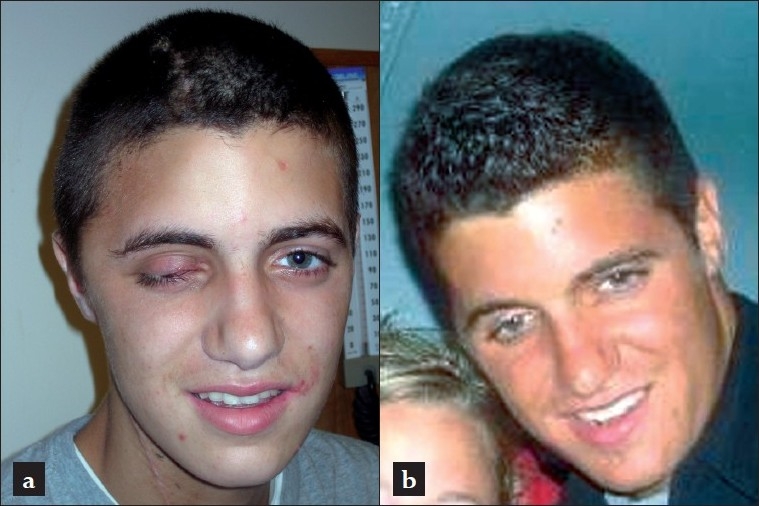
Patient portraits during the postoperative follow-up: (a) at the time of suture removal POD #14 and (b) at 36 months outpatient follow-up.

When the neurosurgery team had completed the critical portion of this procedure, the left hand was explored, surgically debrided and initially reconstructed as a claw with Steinman pins. Meanwhile, a spherical implant was placed by occuloplastics to reconstruct the orbital soft tissue following the enucleation. The postoperative CT scan of the head demonstrated normal post-surgical findings without complications [Figure 6]. The patient was then transferred to the ICU in stable condition. He was extubated on hospital day two, transferred to the floor the same day, and fully mobilized on hospital day three. The patient did not show any focal neurological deficit other than loss of vision on the right side, mild ipsilateral periorbital numbness, and left hand impairment. Due to extensive orthopedic wound care requirements, he was discharged from the neurosurgical service on postoperative day nine. The patient was scheduled to follow-up with outpatient care for planning of his further elective interventions: cranioplasty and staged repair for his upper left extremity and right eye. Of note, the 19-year-old patient was able to attend high-school graduation only 14 days after this incident [Figure 7a].

An uneventful cranioplasty was performed eight weeks after the initial bomb explosion. Outpatient follow up scans have been unremarkable for three years without signs of delayed complications. Clinically the patient remains unchanged. Due to the severe and destructive hand injury, the initially reconstructed claw-fingers had to be amputated at the proximal metacarpal line several weeks later to create a stump with palmar tissue only.

No additional neurological deficits occurred and the patient enrolled to college the year after his accident and successfully graduated in 2009 [Figure 7b]. The patient also was able to master physical labor as field apprentice in a construction firm.

## DISCUSSION

Survived penetrating brain injuries secondary to explosions are very rare in developed countries. However, with the increase in terrorist activity the possibility of a mass casualty from an explosion will rise. Data from terrorist and warfare analyses show that a large portion, up to 36% of blast victims, suffer from head injuries.[12] The approach of minimal acceptable care in such a case was discussed in 2003 by Stein and colleagues.[13] Head trauma patients are on average 9–14 years younger than general trauma patients (34 years, range 2058 vs 43 years, range 25–64), are more likely to be men (73% vs 58%), have more severe injury (ISS median 20 vs 9), and have a higher mortality (25% vs 3%) than those without head injuries.[10] It has been shown that patients with a GCS of 8 and higher, or single lobe brain lesion benefit from early aggressive management[14,16] There is a higher mortality (61% vs 35%) in severe head trauma if treated in nonneurosurgical versus neurosurgical centers. Time is of the essence^16^ and appropriate imaging is a necessity. The correlation between the presence of transventricular or bihemispheric trajectory of a head penetrating object and poor outcome are significant.[8] Besides neurological deficits (depending on the injured area), seizures, CSF leaks, and cerebral infections are the most frequent complications after a penetrating head injury. Infections with a versatile spectrum of causative organisms mainly develop 3–6 weeks after a trauma.[8]

In this case, the patient’s mechanism of injury was consistent with a penetrating traumatic injury and a wound likely containing multiple small remnants and at least one large foreign body. The origin, size, and trajectory of this injury placed the patient at risk for infection at a number of different sites. However, the patient was sufficiently treated with the empiric antibiotic coverage.

In our case, the potentially devastating consequences of this penetrating injury were decreased due to several facts: the projectile did not dislodge large portions of the bone and did not cause fatal bleeding from adjacent vessels. Intracerebrally, it only affected the right temporal lobe (a single site and a noneloquent area of the brain) which also did not lead to any focal neurological deficits after removal of the object and temporal lobectomy. Furthermore, the patient showed very little decline in his level of consciousness after the blast and had a GCS of 15, which is an excellent prognostic factor. Fortunately, after expert emergent care, the patient did not develop any complications.

This case underscores the importance of having a high suspicion for the presence of an intracranial injury and a retained foreign body in the setting of a penetrating head injury, especially in individuals with an unclear mechanism of injury. Aggressive and timely workup as well as expeditious surgical management are crucial in these settings and can generate exceptional outcomes despite a major head trauma.
